# Assessment of Testicular *Lhcgr* mRNA Expression Correlated with Testis and Seminal Vesicle Activities in the Libyan jird (*Meriones libycus*, Rodentia: Muridae) during Breeding Season Compared with Nonbreeding Season

**DOI:** 10.3390/ani11020320

**Published:** 2021-01-27

**Authors:** Radia Boufermes, Mansouria Belhocine, Zaina Amirat, Farida Khammar

**Affiliations:** 1 Biochemistry Department, Faculty of Sciences, Badji Mokhtar University, Annaba 23000, Algeria; 2 Laboratory of Sciences and Technology of Animal Production, Department of Biology, Faculty of Nature and Life Sciences, Abdelhamid Ibn Badis University of Mostaganem, Mostaganem 27000, Algeria; manbelhocine@hotmail.com; 3 Arid Lands Research Laboratory, Department of Population and Organisms Biology, Faculty of Biology, Houari Boumediene University of Sciences and Technology (USTHB), Algiers 16111, Algeria; amiratzaina@yahoo.fr (Z.A.); faridakhammar@gmail.com (F.K.); 4 Department of Biology, Faculty of Sciences, Benyoucef Benkhedda University of Algiers I, Algiers 16000, Algeria

**Keywords:** Libyan jird, mRNA expression, seasons, reproductive cycle, testis *Lhcg* receptor

## Abstract

**Simple Summary:**

The breeding periods of desert rodents should be favorable to the survival of small young, conditioned by the availability of food that occurs in the Libyan jird biotope during the long photoperiod and the high temperatures. The Libyan jird (*Meriones libycus*) were caught in their natural biotope in the Saharan desert in Algeria and showed a seasonal cycle of the testis activity, characterized by the highest peak during spring (the breeding season) and the lowest activity was registered during autumn and winter (nonbreeding season). *Lhcgr* mRNA expression is increased in autumn and decreased in spring. This expression varied in an opposite manner to testicular and seminal vesicle structures.

**Abstract:**

The Libyan jird (*Meriones libycus*, 1823) is a wild desert rodent that is a seasonal breeder species adapted to breed when the environmental conditions can satisfy the energy and hydrous requirements of pregnant and nursing females to ensure that births occur at the most favorable time of the year. We assessed gene expression of testicular luteinizing hormone receptor (*Lhcgr*) correlated to testis activity. The expression of *Lhcgr* was evaluated using quantitative Real Time-Polymerase Chain Reaction (qRT-PCR and the testis activity by a histological method in adult male Libyan jirds during the nonbreeding and breeding seasons. Our results showed that *Lhcgr* mRNA expression increased in autumn during the nonbreeding season and decreased in spring during the breeding season. This expression varied in contrast to testicular structure or function and plasma testosterone levels. These results help to elucidate this desert rodent’s seasonal sexual activity, which is correlated with central regulation.

## 1. Introduction

The framework supporting the gonadotropic axis is composed of different major elements—hypothalamic gonadotropin-releasing hormone (GnRH), pituitary gonadotropins (luteinizing hormone (LH) and follicle-stimulating hormone (FSH)) and gonadal sex steroids [1]. Numerous central and peripheral signals modulate the gonadotropic axis [2]. 

Hence, kisspeptins, and multifunctional peptides are identified that stimulate and regulate GnRH/LH/FSH secretion at puberty and adulthood through the activation of their cognate receptor GPR54. The classical G protein-coupled receptor constitutes an important parameter in the regulation of gonadotropic axis [3,4].

The *FSHr* gene is expressed by the somatic Sertoli cells that control germ cell development. *LhcgR* expression is restricted to the Leydig cells located in the interstitial compartment and is responsible for steroidogenesis in the testis. Data on the sexual activity associated with changes in pituitary gonadotropin expression of several wild mammal species are reported in bear [5], blue foxes [6], rams [7], ground squirrels [8], and roe deer [9]. The Eld’s deer probable existence of endogenous seasonal rhythm operating independently of photoperiod cannot be excluded [10]. 

Photoperiod is an environmental cue, and also factors such as nutritional and social status may influence the reproductive function. Thus, variations in rainfall and temperature in dry deserts are key triggers for early reproduction in small mammals [11].

Most rodents living in arid and semi-arid regions are seasonal breeders and show several variations in testicular activity during the year. Indeed, during the active period, the reproductive organs show considerable growth in weight; structure and ultrastructure are developed and both testicular and plasma testosterone levels increase. The nonbreeding season is typically characterized by a reduction in the size of reproductive organs (seminal vesicle and prostate) induced by a marked reduction in the testicular size and the plasma testosterone level [12,13,14,15].

We previously reported that in the Libyan jird (*Meriones libycus*), a seasonal breeder, *KiSS-1* expression was higher during the breeding season compared to nonbreeding season. Whereas *LHβ* and *FSHβ* expression levels were higher during the nonbreeding season in autumn and varied in an opposite manner with testicular and seminal vesicle weights and with plasma testosterone [16]. It seemed interesting to study the response to LH at the peripheral level and in particular the testicular level, where Leydig cells are the main LH target.

Therefore, the overall objective of our study was to obtain knowledge on the endocrine regulation of seasonal testis maturation in the Libyan jird. Testicular mRNA *Lhcgr* expression was measured in the testis of the Libyan jird during the breeding and nonbreeding seasons. These data were integrated with results on steroid plasma levels, germ cell proliferation and apoptosis in the testis of the same animal. The *Lhcgr* mRNA expression in the testis could be a new parameter to explain the seasonal regulation of reproductive function in the Libyan jird.

## 2. Material and Methods

### 2.1. Animals

Animals were provided from their natural biotope in the Béni Abbès area (30°07′ N, 2°10′ W) in Bechar, Algeria. A total of 10 adult males were captured between September 1 to 10, 2010 and 2017 corresponding to the nonbreeding season, and 10 males were captured between March 10 to 20, 2010 and 2017 corresponding to the breeding season. Adult reproductive condition was evaluated according to body weight (60–140 g), and genital status was assessed during the breeding season when testis and seminal vesicle weights were higher than during other seasons. The animals were placed in individual cages (50 cm in length, 35 cm in width, and 30 cm in height). Animals were kept for approximately 24 h and then euthanized under ketamine anesthesia between 13:00 and 18:00. The left testis was removed immediately, frozen in liquid nitrogen, and stored at −80 °C until assayed, and the right testis and seminal vesicles were used for histology.

### 2.2. Histology

The right testes and Seminal Vesicles (SV) were quickly excised, carefully freed from surrounding fat, weighed, and then fixed in 10% neutral buffered formalin for 24 h. Testes and SV were dehydrated in a series of increasing degrees of ethanol (70%, 95%, and 100%) and cleared in cyclohexane. After 12 h of impregnation in paraffin in an incubator at 60 °C, testes were embedded in paraffin. The paraffin-embedded tissue samples were sectioned at a 5 μm thickness (100 section /testis/animal) with a Leitz 1512 rotatory microtome (Marshall Scientific, Hampton, VA, USA; SNH Southern New Hampshire, Nashua, NH, USA). After dewaxing and rehydration, the sections were stained either with Masson’s trichroma or Azan of Heidenhain, Van Gieson’s trichroma, or Sirius red and then they were dehydrated in ethanol. The slides montage was performed with the use of a neutral resin Euckitt (Sigma-Aldrich Chemie, GmbH Export Department, Taufkirchen, Germany). The slides were observed at different magnifications (100×, 400×, and 1250×) under a light microscope, and a Zeiss photomicroscope (Carl Zeiss Microscopy GmbH, Jena, Germany) was used to take microphotographs. For morphometric measurement, the diameter of seminiferous tubules (µm) with round or nearly round profiles were evaluated for each animal on 100 seminiferous tubules per animal, and the mean ± standard deviation (SD) of the diameter was determined by taking the average of two diameters—SI and S2 (perpendicular to one another). Height (µm) of seminal vesicle epithelial cells and height (µm) of their supranuclear zona were measured on six slides from each animal and 10 randomly selected fields from each slide were calculated. The microscope fields were examined under a 40× objective. Morphometric parameters were calibrated using manual micrometry [17].

### 2.3. mRNA Extraction and Reverse Transcription 

Total RNA was extracted from a single testis using TRIzol reagent (Invitrogen, Carlsbad, CA, USA) following the manufacturer’s instructions. The total RNA concentration was determined by spectrophotometry at 260 nm. Two micrograms were reverse transcribed in a reaction mixture (40 µl) containing 100 U of Moloney murine leukaemia virus reverse (Amersham Pharmacia Biotech, Orsay, France), reaction buffer (Amersham, Pharmacia Biotech, Orsay, France), 0.2 mM deoxynucleotide triphosphates (Promega, Charbonnières, France), 50 pmol oligo (dT) primers (Invitrogen, Life technologies, USA) and 20 U of ribonuclease inhibitor (Promega, Charbonnières, Cergy Pontoise, France).

### 2.4. Polymerase Chain Reaction (PCR)

With the alignment of the *Lhcgr* sequences obtained from the following different species and accession numbers (Mongolian gerbil (AB571125); Syrian hamster (AB571126); rat (NM_181692.1), mouse (NM_178260.3), and opossum (NM_001144132.1), we identified the core sequence from which several sets of primers were designed to facilitate amplification of the specific polymerase chain reaction (PCR) products for Libyan jird—*Lhcgr* and β-actin. Primers were designed using Primer Express software ver. 3.0 (Applied Biosystems, Foster City, CA, USA) (Table 1). The conditions were 50 °C for 60 min and 95 °C for 15 min, followed by 35 reaction cycles at 94 °C for 15 s, 55.4 °C for 30 s, and 72 °C for 30 s each. The amplified fragments were separated by electrophoresis in 1.5% agarose gel and visualized by ethidium bromide staining.

### 2.5. Sequencing

PCR products were purified and sequenced using a CEQ DTCS Quick Start Kit on a DNA sequencer (Beckman Coulter, Fullerton, CA, USA).

### 2.6. Quantitative Real-Time PCR

After identification of the specific Libyan jird cDNA sequences *Lhcgr* and β-actin, we designed new primers on two different exons for the quantitative real-time PCR analysis using the Primer Express software (Table 2). Relative levels of total *Lhcgr* mRNA were examined by real-time PCR using an ABI Prism 7000 Sequence Detector System and the SYBR Green Universal PCR Master Mix, according to the manufacturers’ recommendations (Applied Biosystems, Courtaboeuf France). Briefly, samples were heated for 10 min at 95 °C, followed by 40 cycles or 45 cycles of 15 s at 95 °C and then 1 min at 60°C in a total volume of 25 µl. The PCR was performed in duplicate, and a reagent blank, which was prepared using the Retro Transcriptase blank was included on each plate to detect contamination by genomic DNA. The reaction products were resolved in a 1.5% agarose gel and visualized with ethidium bromide to determine the lengths of the amplified DNA fragments. The β-actin gene was used as an endogenous control to normalize the initial RNA level. The ΔΔCt method [18] was used to estimate the relative amounts of testicular *Lhcgr* mRNA from each season compared with those of the internal standard (β-actin), and the values were compared.

### 2.7. Statistical Analysis

All data are presented as the mean ± standard error. Differences between means were evaluated using the Student’s t-test. We used GraphPad Prism (version 5; GraphPad Software Inc., San Diego, CA, USA) to determine the differences in the various parameters between seasons. Values of *p <* 0.05 were considered to be significant.

## 3. Results

### 3.1. Seasonal Changes of Testes Structure and Seminal Vesicles

**Figure 1:**
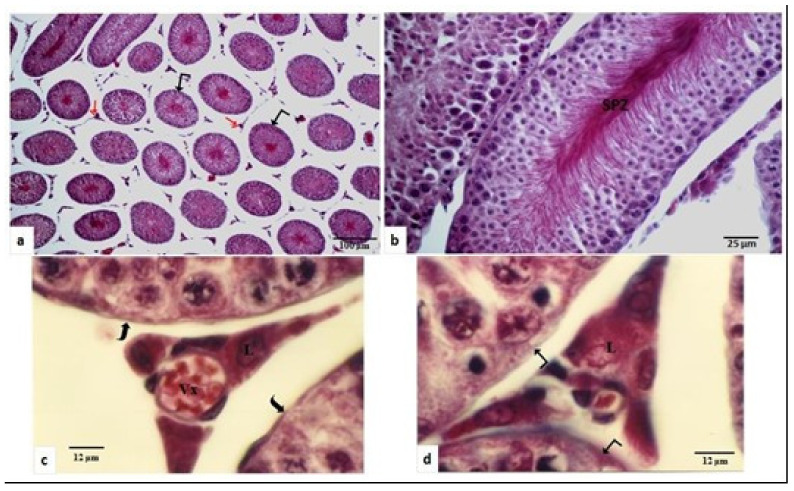
Testicular structure of *Meriones libycus* in the breeding period (spring and early summer). (**a**) Seminiferous tubules diameter (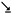) is high (700 µm ± 806 µm) with regular outline Leyding cells (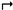) are observed in the inter-tubular space. (**b**) The spermatogenesis is very active and numerous spermatozoa (SPZ) are accumulated in the lumen of the seminiferous tubule. (**c**) Leydig cells (L) are large and arranged around blood vessels (Vx), and the basement membrane (⊇) is regular, intact, and thin. (**d**) Leydig cells (L) are located in the vicinity of the seminiferous tubules (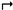). (**a**): ×100; (**b**): ×400; (**c**,**d**): ×1250.

In the breeding season (spring), under light microscopy, the diameter of the seminiferous tubules was large (700 µm ± 8.6 µm), with a regular form and intact basement membrane (Figure 1a). Spermatogenesis was very active, and many spermatozoids were observed in tubule lumens (Figure 1b). In the inter-tubular space bulky dispersed Leydig cells were observed, which were located near the walls of the seminiferous tubules (Figure 1d) or contiguous to the blood capillaries (Figure 1c). The cells had rounded or oval nucleus occupying the center of the cell (Figure 1c,d).

**Figure 2:**
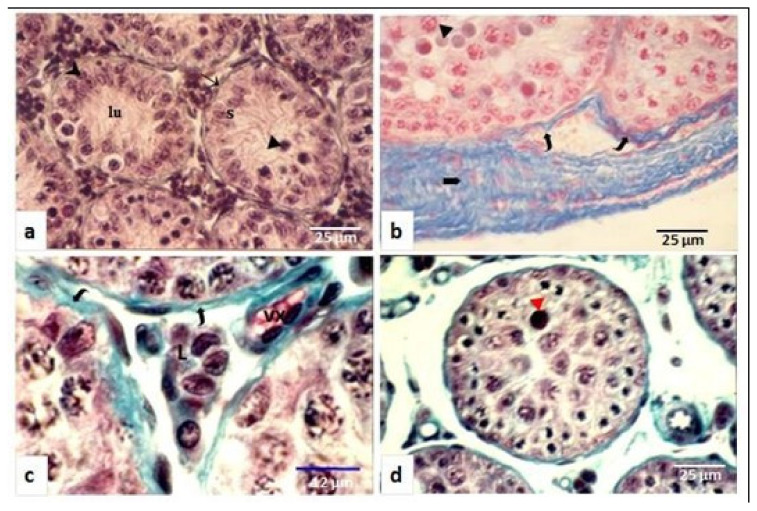
Testicular structure of *Meriones libycus* in the nonbreeding period (late summer, autumn, and late winter). (**a**) Seminiferous tubules (→) with a reduce diameter (300 ± 6.4 µm) and an irregular contour, spermatogonia (▯) remain close to the basement membrane; the lumen (lu) does not contain any sperm, Sertoli cell nuclei (S) are moved away from the basement membrane, and some degenerated germ cells are observed (►). (**b**) The basement membrane (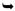) is thick and pleated and the tunica albuginea (➨) is greatly dense. (**c**) Leydig cell (L) are of greatly reduced size and seminiferous tubules are bordered by a deep basement membrane (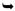). (**d**) Spermatogenesis stopped at the stage of spermatogonia and tubular lumen completely disappeared. (**a**,**b**,**d**): ×400; (**c**):×1250.

During the nonbreeding period (autumn), the diameter of seminiferous tubules decreased (300 µm ± 6.4 µm) significantly, (*p* < 0.001), and the lumen was reduced or sometimes absent (Figure 2a,c). Sperm was absent and spermatogenesis was stopped at the spermatogonia stage in most observed tubules, with spermatogonia located near the basement membrane that became thick and pleated (Figure 2a–c). Sertoli cells were numerous, and their nuclei with an oval or round shape were far from the basal lamina (Figure 2a). In the inter-tubular space, very small-sized Leydig cells were grouped (Figure 2b).

**Figure 3:**
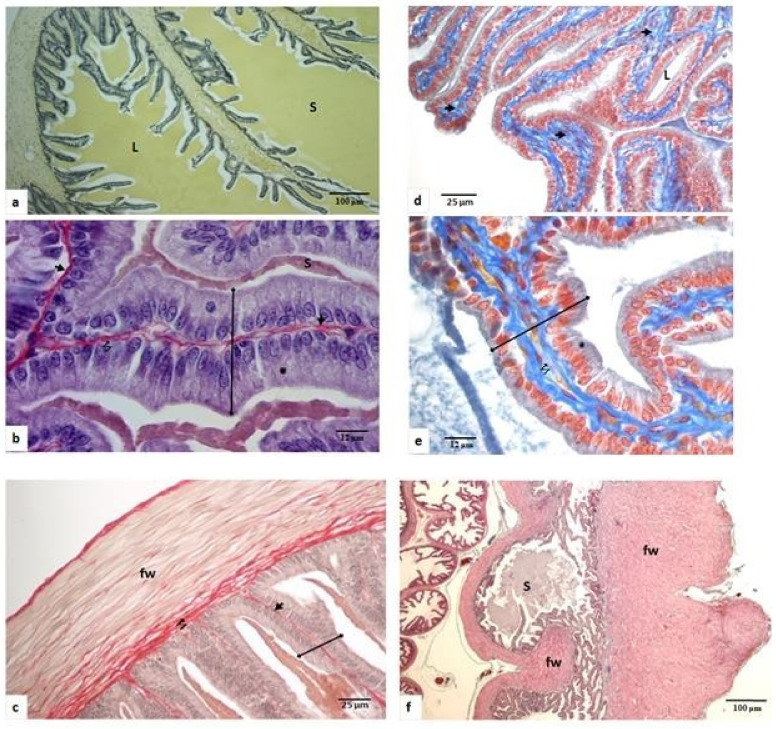
Histological seasonal changes of the seminal vesicle of the Libyan jird (*Meriones libycus*). (**a**–**c**) In the breeding period (spring and onset of summer). (**d**–**f**) In the nonbreeding phase (end of summer, fall, winter). Secretion (S), epithelial folds (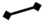), epithelial fold axis (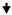), supranuclear zona of epithelial cells (*), lumen (L), connective fibers (↯), fibromuscular wall (fw). Staining: (**a**) Van-Gieson; (**d**,**e**) Azan of Heidenhain; (**b**,**c**,**f**) Sirius red. (**a**,**f**) ×100; (**b**,**e**) ×1250; (**c**,**d**) ×400.

In the breeding season, the seminal vesicles were abundantly filled with secretion (Figure 3a). They had numerous developed epithelial folds with a narrow axis where very few collagen fibers were housed (Figure 3b). They were bordered with a thin fibromuscular wall and separated from the epithelium by some sparse connective fibers (Figure 3c). The cylindrical epithelium was constituted with cells measuring 23 µm ± 1.25 µm in height with an abundant supranuclear cytoplasm without any specializations measuring 13 µm ± 0.8 µm (Figure 3b).

During sexual quiescence, the seminal vesicles were strongly regressed, and in the widened axis of the epithelial folds was found a hypertrophied connective tissue (Figure 3d,e), and the fibro-muscular wall became irregular and well developed (Figure 3f). The epithelium was atrophied and was made up of cuboidal epithelial cells of only 11.6 μm in height, with a reduced supranuclear zone measuring only 3.7 µm ± 0.25 μm (Figure 3e).

### 3.2. Seasonal Changes in Relative mRNA Expression of Lhcgr

**Figure 4:**
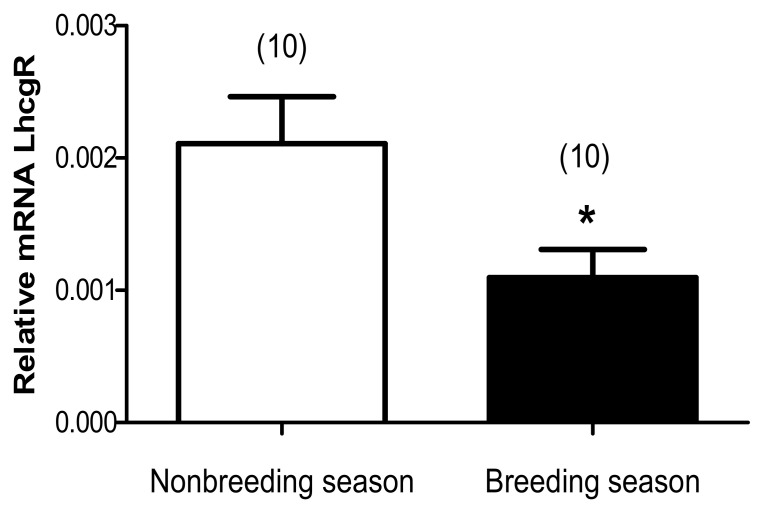
Seasonal changes of testicular LhcgR expression relative to the internal control, β-actin expression in Libyan jird (*Meriones libycus*) live-trapped in the field in the Beni-Abbes area (Algeria). Numbers in brackets represent the number of samples. Significant differences are designed by * at *p* ≤ 0.05 versus between seasons.

The evaluation of the expression of *Lhcgr* mRNA expression was carried out on the tissue of the testis using a quantitative real-time PCR method. The relative *Lhcgr* mRNA expression level was higher during the nonbreeding season (2.10.10^–3^ ± 0.35.10^–3^) than that during the breeding season (0.92.10^–3^ ± 0.15.10^–3^), (*p* < 0.05) a change of 48% (Figure 4) between the two seasons. 

## 4. Discussion

The adult male Libyan jird (*Meriones libycus*) exhibits a seasonal reproductive cycle, with a period of reproductive quiescence during autumn and winter and a period of reproductive activity during early spring until early summer [15]. In this study, the testis was very active in the spring (breeding season) and was characterized by seminiferous tubules with a large diameter and a wide lumen containing many spermatozoa as in the Syrian hamster [19]. Gross Leydig cells were housed in the inter-tubular compartment reflecting a significant testosterone synthesis. From late summer to late winter (nonbreeding season), the diameter of the seminiferous tubules was reduced, and the lumen was significantly diminished and was free of spermatozoa. Spermatogenesis was stopped at the stage of spermatogonia and Sertoli cell nuclei have moved away from the basement membrane. By contrast, in the sand rat, *Psammomys obesus*, which is in testes atrophy during spring, summer, and autumn, and the Syrian hamster, which undergoes gonadal regression in autumn and remains reproductively quiescent throughout winter, spermatogenesis is arrested at the spermatocyte stage [19,20].

These morphological characteristics reflected inhibition of spermatogenic and steroidogenic activities of the testis and correlated with changes in total testicular androgens and plasma testosterone levels [16]. In the viscacha (*Lagostomus maximus*), the testis, which is in the active phase in the summer and the nonbreeding period in the spring, showed similar morphological seasonal variations [21]. Hence, high levels of circulating androgens may exert negative feedback on FSHβ synthesis and release. This effect may also stop spermatogonial proliferation and start the final maturation phase. Histologically, the seminal vesicle is related to testis seasonal activities. The latter was of elevated weight and produced an abundant secretion that filled the lumen and possessed a highly active structure with numerous epithelial folds, and epithelial cells with a large granular supranuclear zone. During gonadal regression, seminal vesicles showed signs of reduced functional activity. These were characterized by a few epithelial folds formed by an epithelium of smaller height and have an enlarged axis where were housed a hypertrophied connective tissue. Knowing that the testosterone content is low during the gonadal regression, our histological results show a significant correlation between the secretory activity of the seminal vesicle and the androgenic activity of the testes. Similar findings were reported in the viscacha (*Lagostomus maximus*) seminal vesicles and prostate [22,23].

Our results showed that *Lhcgr* mRNA expression increased in autumn during the nonbreeding season and decreased in spring during the breeding season. The histological results of the testes and seminal vesicles follow the same profile as the plasma testosterone previously observed [16], and the contents vary in phase opposition compared to *Lhcgr* mRNA expression. These peripheral data will allow us to understand the regulation of reproductive function in the Libyan jird and will help to elucidate this desert rodent’s seasonal sexual activity, which is correlated with hypothalamic-pituitary regulation. Indeed, in our previous study, *LHβ* mRNA expression, and its cognate *Lhcgr* mRNA were less complex considering upregulation of both genes during the nonbreeding season and downregulation during the breeding season. The late maturational process is considered to be more dependent on *LHβ* expression than on *KiSS-1* expression, which is apparently upregulated [16]. The direction of variation of *LHβ* and *FSHβ* that are opposite of their mRNA expression would be predicted by variation in *KiSS-1* expression [16].

Wang et al. [24] performed an immunohistochemical study and found strong expression of kisspeptin in 5- and 15-week-old mice; then these authors [24] proposed that kisspeptin expression in Leydig cells might be correlated with the maturation of Leydig cells or the development of the testes during puberty. A robust level of kisspeptin at postnatal day 28 correlated with the pubertal onset, and using immunohistochemical analysis, these authors located kisspeptin in Leydig cells [24]. Additionally, their data indicate clearly that LH is involved in regulating levels of Leydig cell kisspeptin [25]. Notably, gonadectomy results in elevated LH but reduces serum kisspeptin levels [25]. A recent study concluded that kisspeptin has no role in testicular regulation related to testosterone and inhibin release but may have other roles in testicular regulation [26]. According to our data, *Lhcgr* expression could be implicated in testicular kisspeptin expression. This regulation may be caused by pulsated GnRH, which remains unknown in this species. This seasonal variation of reproductive activity in a desert rodent is similar to that observed in red deer stags (Cervus elaphus). In this species, the LH levels are maximal in August during the phase of testicular redevelopment, whereas the testosterone levels are maximal from September to November, corresponding to the time of peak testicular activity and the mating season. Castrated stags had higher plasma levels of LH than those of the intact stags at all times of the year, and no clear seasonal cycle in LH levels was observed in this species [27]. In bears (*Ursus thibetanus japonicus*), significant variations in plasma testosterone and inhibin concentrations and changes in FSH concentrations preceded those of these hormones with a similar tendency [5]. Hormones started to increase during dinning and achieved the highest values at the end of the recrudescent phase for FSH and in the active phase for testosterone and inhibin. These changes in hormone concentrations were followed by testicular growth. In situ hybridization analysis revealed that *FSH* and *LH* receptor mRNAs are possibly expressed in Sertoli cells and Leydig cells, respectively, as they are in other mammals. However, neither plasma LH concentration nor testicular gonadotropin receptor mRNA expression levels varied significantly among the sampling months. These results suggest that FSH, inhibin, and testosterone have roles in testicular activity in male bears [5].

In adult male hedgehogs, the seasonal variations in gonadal and pituitary activities are in parallel during the year [28,29]. The reactivation of reproduction occurs from the beginning of winter (low and increasing daylengths) in spite of low temperatures, the involution is regular at the end of summer when day lengths are decreasing, and temperatures remain high [30].

In autumn and winter, the regular spontaneous recrudescence of pituitary/testicular activity occurs after several months of rest. This phenomenon could be a state of photo refractoriness, which is broken later in autumn, or a negative feedback control from increased levels of LH in autumn and corresponding to the development of refractoriness to short days at the central level. This hypothesis is consistent with similar results obtained in hamsters [31,32].

In the impala (*Aepyceros melampus*), the increase in testosterone secretion during the hot season (activity) is associated with increased susceptibility to testicular LH receptors from an increase in circulating gonadotropins or with pituitary sensitivity to GnRH. In this species, LH is critical to start the steroidogenic activity of the testis, but for some species, testosterone and FSH are important and occur later during the maximum activity cycle to ensure spermatogenesis [33].

In rams, peak LH levels are associated with the increase in testicular LH receptor concentration combined with elevated LH levels. Additionally, the peak concentrations of FSH occur later, after the LH peak, and are parallel to the testosterone cycle and sperm production. The mechanisms to explain these differences between the expressions of *FSHβ* and *LHβ* remain unknown [7]. GnRH or inhibin may be involved in the regulation of the reproductive activity of the Libyan jird, although such regulation has not been investigated. In the sand rat (*Psammomys obesus*), a short day breeder, plasma LH concentration increases in early summer and that intravenous administration of GnRH (200 ng/100 g of body weight) fails to elicit significant season-dependent changes in LH release, whereas the increase in plasma testosterone was maximum in June–July and low between November and March–April. The summer seasonal onset of the testicular endocrine activity of the sand rat is due to increases in both in LH release and testes sensitivity to gonadotropin [34]. Other studies showed that GnRH is involved in the regulation of pituitary gonadotropin secretion by a system that depends on frequency pulsations. Thus, rapid pulsations of GnRH promote increased expression of the *LHβ* messengers and therefore its secretion; conversely, weak pulsations increase transcription of *FSHβ* mRNA [35,36].

In several species, the increase in serum FSH occurs before the beginning of the breeding season and is often associated with an increase in testicular weight rather than the maintenance of spermatogenic activity [37,38]. In terms of seasons, the body temperature is a factor triggering the increase in testosterone during the hot season [39]. The breeding periods of desert rodents should be favorable to the survival of small young, which are conditioned by the availability of food that occurs in the Libyan jird biotope during the long photoperiod and high temperatures. The study of thyroid hormones could further explain the seasonal regulation of testicular function. Indeed, in adult male European hedgehogs, which were studied in the field, the plasma levels of both testosterone and thyroxin exhibited a marked annual cycle with a minimum in autumn and a maximum during spring and summer. The winter resumption of the testicular activity started to decline simultaneously in August. The correlation between the two hormones was less close from May to July. Similar results were reported in other species [40,41].

## 5. Conclusions

Our study demonstrates a complex interplay between the testicular activity and gonadal *Lhcgr* in the Libyan jird. On one hand, seasons can influence *LHβ*/*Lhcgr* expression, which is presumed to be essential for initiating reproductive activity during the long day in the Libyan jird. On the other hand, during the same period, high weights of gonadal tracts demonstrated testicular activity. The seasonal variation of testis activity is apparently dependent on the species and on the environmental cues that modulate the seasonal physiology to a large array of endocrine and metabolic signals.

## Figures and Tables

**Table 1:** Oligonucleotid primers used for amplification of the testicular cDNA *LhcgR* based on *Rattus norvegicus* (Accession number NM_012978).

Primers	Sequence 5′→3′	Product Size ^1^(bp)	Exons (E)
*LhcgR*	F: TGC ACA GTG GAG CCT TCC R: ATT CCG CCA TCT TTG AGG	329	F: E1 R: E2

^1^bp: base pairs; F: forward; R: reverse.

**Table 2:** Sequences and exonic localization of the oligonucleotides primers of *LhcgR* and *β-actin* used for real-time polymerase chain reaction (PCR) analysis on partial sequence of *Meriones libycus* cDNA *LhcgR*.

Primers	Sequence 5′→3′ ^1^(bp)	Product Size ^1^(bp)	Exons (E)
*LhcgR*	F: GCT GC GCT TTT AGG AAT TTG R: CCA AAC AAT GTG AAA GCA CA	86	F: E1 R: E3
*β-actin*	F: ATG TTG CCC TGG ACT TTG AGR: CCT CTC ATT GCC AAT GGT GA	151	F: E3 R: E4

^1^bp: base pairs; F: forward; R: reverse.

## Data Availability

The data presented in this study are available on request from the corresponding author.
